# Vinpocetine-based therapy is an attractive strategy against oxidative stress-induced hepatotoxicity in vitro by targeting Nrf2/HO-1 pathway

**DOI:** 10.17179/excli2021-3463

**Published:** 2021-03-04

**Authors:** Noha Abdelmageed, Wael Ahmed-Anwar Twafik, Abdel-latif Seddek, Samy Abdel-Raouf Fahim Morad

**Affiliations:** 1 Department of Pharmacology, Faculty of Veterinary Medicine, Sohag University, Sohag, Egypt; 2 Department of Biochemistry, Animal Health Research Institute (AHRI), Qena branch, Qena, Egypt; 3 Forensic Medicine and Toxicology Department, Faculty of Veterinary Medicine, South Valley University, Qena, 83523, Egypt; 4 Department of Pharmacology, Faculty of Veterinary Medicine, South Valley University, Qena, 83523, Egypt

**Keywords:** Vinpocetine, oxidative stress, GSH, ROS, hepatotoxicity, H2O2, Nrf2, Keap1

## Abstract

Vinpocetine (Vin), a synthetic-derivative of Vincamine, monoterpenoid indole alkaloid, has been reported to have various medicinal benefits. The purpose of our study was to investigate the pivotal role of “nuclear factor erythroid 2-related factor-2” (Nrf2)-mediated antioxidant protection of Vin against H_2_O_2_ and paracetamol (APAP)-induced liver toxicity. For this purpose, a normal human hepatic cell line (L02 cells) was incubated with cytotoxic concentrations of H_2_O_2_ or APAP in the presence or absence of Vin. To evaluate the responses, MTS Cell Viability assay, immunoblotting, biochemical assays, and molecular docking approach were used. Viability analysis showed that treatment of L02 cells with Vin prevented the cytotoxicity induced by H_2_O_2_ and APAP. It was evidenced by the fact that Vin dumped H_2_O_2-_ and APAP-cytotoxicity and reactive oxygen species (ROS) generation. The immunoblotting analysis shows that Vin increased Nrf2 expression along with the expression of target protein, heme oxygenase-1 (HO-1), and increased intracellular glutathione (GSH) level. Interestingly, we found that Vin could protect the protein expression-level of Nrf2, which indicated the prospective interaction between Vin and Keap1 protein. Additionally, molecular docking-study revealed that Vin competed with Nrf2 for Keap1-binding site, with hydrogen and stearic interactions. Collectively, Vin effectively protects against H_2_O_2_ and APAP-induced cytotoxicity via executing Nrf2-mediated restoration of antioxidative/oxidative balance. Meanwhile, Vin interrupts protein-protein interaction between Nrf2 and Keap1, which might also contribute to decrease Nrf2 degradation and stabilize protein expression. Thus, Vin-based adjuvant therapy may represent a smart drug regimen to mitigate drug-induced oxidative stress and liver injuries.

## Abbreviations

Nrf2, Nuclear factor erythroid 2-related factor 2

Keap1, Kelch-like ECH-associated protein 1

GSH, Glutathione

ROS, Reactive oxygen species

APAP, Paracetamol (or acetaminophen)

PDE1, phosphodiesterase 1

NAC, N-acetyl cysteine

FBS, Fetal Bovine Serum

## Introduction

At cellular level, cells are consistently subjected to Reactive oxygen species (ROS) and electrophiles, from both endogenous biological activities and also from exogenous resources. These reactive insults not solely destroy the cellular macromolecules, such as nuclear acids, protein, and lipids, but also trigger harmful oxidative signaling (Costantini, 2019[5]). However, correct quantity of ROS is essential in signal transduction, so a fine-tuned antioxidant-system is fundamental and critical for the cells.

Nrf2 is an essential transcription factor that controls cellular antioxidant-responses. It has been considered as a key regulator of cellular defense system to mitigate ROS and electrophile harmful effects. By binding to ARE, Nrf2 triggers the transcription of essential genes that are involved in the glutathione-based and thioredoxin-based antioxidant-defense systems (Dodson et al., 2019[6]; Unoki et al., 2020[18]).

Kelch-like ECH-associated protein 1 (Keap1) is considered “the switch of Nrf2 protein activation” in response to cellular oxidative stress. Keap1 acts as a protein-adapter in the “cullins3 (Cul3)-based E3 ligase-complex”, that is interacting with Nrf2 protein by its “Kelch-repeat-domain”, and with Cul3 (Bresciani et al., 2017[4]). Under physiological conditions, cytoplasmic-Nrf2 is sequestered by Keap1-dimer and subsequently ubiquitinated by “Keap1−Cul3 E3 ligase”, leading to the degradation of Nrf2 protein by the “ubiquitin-proteasome degradation” and this process is called “Keap1-mediated proteasomal degradation”. Under stress conditions, the amino acid-cysteine-residues (the reactive residues) in Keap1 protein are covalently modified by increased ROS and electrophiles, resulting in the conformational/structural changes of Keap1 protein that inactivates “the Keap1− Cul3 E3 ligase” (Bresciani et al., 2017[4]; Silva-Islas and Maldonado, 2018[16]; Unoki et al., 2020[18]). Therefore, the newly synthesized Nrf2-protein can get rid of “Keap1-dependent degradation”, leading to increase the free Nrf2 protein level that will be translocated into nucleus to initiate the transcription and synthesis of newly antioxidant target genes, such as HO-1. The sufficient-protein levels and timely Nrf2-activation is pivotal for cells to antagonize stress insults, where the inadequate level of Nrf2 has been implicated as pathogenic-executive players for myriad inflammatory and stress-related disorders (Unoki et al., 2020[18]; Dodson et al., 2019[6]; Silva-Islas and Maldonado, 2018[16]). Increasing Nrf2 activity and protein synthesis could have potential usages in various stress insults-related diseases that may involve in neuronal, respiratory, hepatic, and gastrointestinal disorders. 

**Figure 1:**
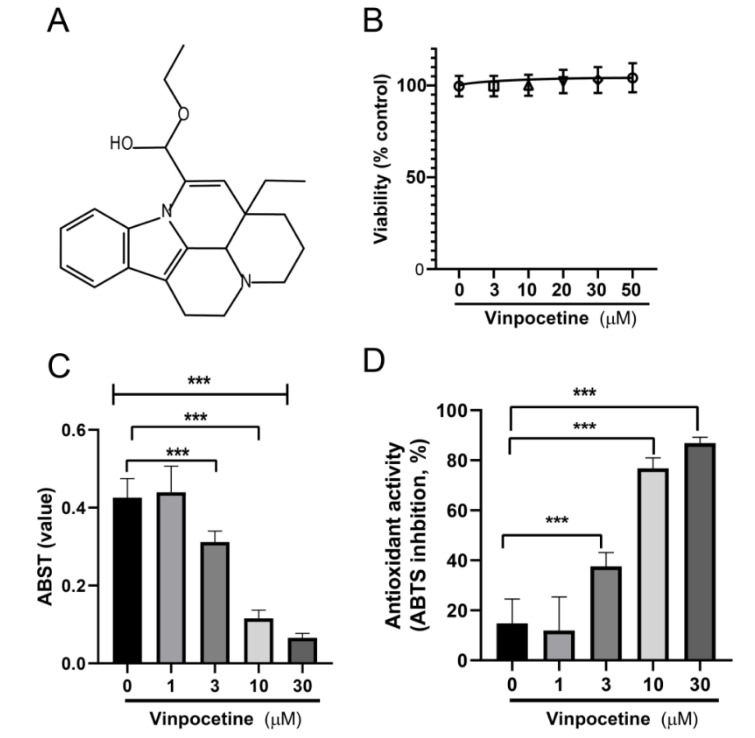
Effects of Vinpocetine on the cell viability of L02 cell and detection of antioxidant activity of Vinpocetine. A) Chemical structure of Vinpocetine. B) The cytotoxic effects of Vinpocetine (various concentrations) on L02 cells were determined using a MTS viability assay after 24 hrs treatment. C) Detection of antioxidant activity of Vinpocetine by ABTS radical scavenging activity assay. D) Antioxidant activity (%). Data are presented as means ± SD. *** p < 0.0001 versus control group

Vinpocetine (ethyl apoVincaminate, Vin) is produced by minor modification of the Vincamine (Figure 1A(Fig. 1)), an alkaloid active principle extracted from the “periwinkle plant”, *Vinca minor*. Vin was originally marketed in 1978 as a therapy for cognitive impairments, such as stroke, amnesia dementia, delirium, and memory conflicts. Currently, different kinds of Vinpocetine-containing memory pills such as Vinporal (AMIRYA, Cairo, Egypt), Cavinton (Gedeon Richter, Hungary) and Intelectol (Memory Secret, Miami, FL, USA) are used worldwide as food additives. Up to now, Vin has a therapeutically safe profile without any significant unwanted effects or toxicity. Vin has myriad pharmacological effects: 1) it acts as a vasodilator leading to improve cerebral blood flow; 2) enhances cerebral glucose and oxygen uptake, that lead to a significant improvement in brain metabolism and 3) stimulates production of ATP in the neuron (Al-Kuraishy et al., 2020[2]; Bereczki and Fekete, 2008[3]; Patyar et al., 2011[14]; Zhang and Yang, 2014[19]; Zhang et al., 2018[20]), as well as serving as a potent anti-inflammatory agent (Medina, 2010[12]; Zhang and Yang, 2014[19]; Zhang et al., 2018[20]). Vin also has multiple downstream cellular targets, such as, 1) -phosphodiesterase 1 (PDE1), 2) -voltage-dependent Na+ channels (Zhang et al., 2018[20]), and 3) -IκB kinase (IKK) (Medina, 2010[12]; Zhang et al., 2018[20]; Jeon et al., 2010[7]).

In this study, we investigated the possible novel role of Vinpocetine as a potent Keap1−Nrf2 inhibitor and also show therapeutic potential of Keap1−Nrf2 inhibition against APAP-induced and H_2_O_2 _-induced cytotoxicity.

## Materials and Methods

### Cell lines

Normal human hepatic cell line (L02 cells) was purchased from the American Type Culture Collection (ATCC, Manassas, VA), and maintained in RPMI 1640 medium (containing 10 % (v/v) fetal bovine serum (FBS), penicillin of 100 U/mL, and streptomycin of 100 g/mL).

### Viability assessment (MTS)

Cell viability protocol was performed as previously described (Morad et al., 2013[13]) using a commercially available CellTiter-96 proliferation assay (MTS) kit (Promega, Madison WI). In brief, L02 cells were cultured and plated in 96 well plate with standard full RPMI 1640 medium supplied with 10 % FBS, then incubated in a 5 % CO_2_-incubator at 37 °C. After 24 hrs, cells were treated with different compounds in 200 μl media. Vinpocetine was dissolved in DMSO (3.5 mg/ml, 10 mM) and then diluted with culture medium to various concentrations (0 to 30 M) for following tests (the final concentration of DMSO was 0.05 %). After 24 hrs, cells were subjected to standard protocol for MTS and measured colorimetrically at 490 nm wavelength using a microplate reader.

### ABTS Radical-Scavenging Assay

The antioxidant activity of Vinpocetine was measured by ABTS (2,2-azino-bis (3-ethylbenzothiazoline-6-sulfonic acid) diammonium salt free radical) assay according to the instruction of ABTS Antioxidant Assay Kit (Sigma-Aldrich, CS0790-1KT). The cells were treated with escalated concentrations of vin (0 up to 30 μM) for 24 hrs, then the antioxidant power was measured colorimetrically. The colorimetric assessment was performed at 734 nm absorbance, using Synergy-microplate reader (Synergy H1MF, BioTek, Winooski, VT, USA). The obtained results were expressed as the mean ± standard deviation.

### Intracellular ROS measurement

The intracellular ROS level was evaluated by fluorescent DCFDA Assay Kits. L02 cells (1×10^5^ cells/mL) were plated into 96-well plates and cultured for 24 hrs. Afterwards, cells were subjected to H_2_O_2_ (0.5 mM) or APAP (5 mM) in absence and presence of Vinpocetine (20 μM) or N-acetyl cysteine (10 mM, NAC). After 24 hrs, cells were washed with warm PBS and stained with DCFH-DA (10 μM) for 20 min at 37 °C. Finally, the cells were rinsed using 200 μL PBS (3X) and the fluorescence intensities of DCF-dye were measured using a VICTRO3 fluorescence-plate reader (VICTRO3, PerkinElmer, MA, USA), with wavelength 485/535 nm (excitation/emission wavelength).

### Detection of intracellular reduced GSH

Intracellular GSH concentration was measured using a commercially available glutathione detection kit (Biodiagnostic, Egypt). The cells were handled and treated as described under “Intracellular ROS measurement”. At the indicated incubation time, cells were harvested by trypsin and washed with PBS (3X). Then, the glutathione level was measured colorimetrically at 420 nm wavelength using microplate reader (SpectraMax M5, MD, CA, USA).

### Western Blot

Western Blot was performed as described previously (Abdelmageed et al., 2017[1]). In brief, after the treatment regimen, cells were washed using ice cold PBS, then were lysed using RIPA lysis buffer (Cell Signaling Technology Inc., Danvers, USA), containing a protease inhibitor (1 mM PMSF) and phosphatase inhibitors. Protein concentration was determined using BCA method. Thirty microgram proteins (30 µg) from each sample were loaded into gradient-NuPAGE gels (Invitrogen, 4-12 % Bis-Tris gels) and transferred onto a PVDF membrane. The membranes were blocked using 5 % skim-milk, then were incubated with primary antibodies against Nfr2 and NO1 (Cell Signaling Technology Inc., Danvers, USA, dilution 1:3000). Eventually, the membranes were incubated with appropriated horseradish peroxidase (HRP)-conjugated secondary antibody (dilution 1:5000). B-actin was used as loading reference. The band intensities were quantified using imageJ software. 

### Molecular docking

The molecular docking was performed using Autodock software. Autodock (version-4.2.6) was used to determine the possible binding mode of Vinpocetine at the active-binding site of Keap1 and to detect the most appropriated residues for binding. For ligand preparation, the structure of Vinpocetine was downloaded from PubChem with CID: 443955, and finally optimized to minimal energy using Maestro-Ligprep-module (LigPrep, Schrödinger, LLC, New York, NY, 2020). LigPrep-module was utilized to ensure optimization of Vinpocetine geometry, to remove structural problems, and to generate Vinpocetine structural-variants. LigPrep generated low energy Vin 3D structure with optimized chirality, and the Vin was minimized using OPLS2005 force field. For preparation of Protein, the crystal structures of Keap1 (PDB ID code 4L7B), with determined resolution of 2.41, was obtained from the Protein Data Bank (https://www.rcsb.org/). In this study, the protein-preparation-Wizard (PrepWizard) in Maestro was utilized to prepare the Keap1 protein structure for docking. The ultimate goal of this step is to guarantee the correct protein structure, proper assignment of bonds and bond orders, to add missed hydrogens, to complete in any missing loops or side chains of the protein. Furthermore, the protein 3D structure of 4L7B was restrained-minimization using the OPLS2005-force field. During this minimization process the heavy atoms in 4L7B structure are restrained to relieve torsional strain with a harmonic potential of 25 kcal mol−1 Å−2 and hydrogens remain unrestrained. The visualization of Vinpocetine-Keap1 was performed by using BIOVIA Discovery Studio Visualizer v.4.5 (Accelrys).

### Statistical analysis

For statistical analysis, results are presented as mean ± SD. Data were analyzed using GraphPad Prism-8 software (La Jolla, CA., One-way ANOVA and a Tukey's post-hoc test) to estimate the differences between three or more groups. P value less than 0.05 (P<0.05) was considered statistically significant.

## Results

### Cytotoxicity of Vinpocetine on the L02 cell line and ABTS radical scavenging activity 

First, the safety profile of Vin in the L02 cell line was determined by using MTS commercial kits. As illustrated in Figure 1B(Fig. 1), no cytotoxicity effects were observed in cells treated with Vin up to 50 μM, for 24 hrs incubation period. Therefore, in all subsequent experiments, Vin was used at a concentration between 1 to 30 μM.

ABTS reagent produces green color (ABTS·+) under the appropriate antioxidant condition, and this ABTS·+ production is inhibited with the antioxidants. Thus the total antioxidant capacity of the cells treated with or without Vinpocetine can be determined and calculated by measuring the sample absorbance of ABTS·+ at 734 nm. The capacity of Vinpocetine to scavenge ABTS·+ is shown in Figure 1C-D(Fig. 1). As illustrated in Figure 1C-D(Fig. 1), Vin exhibited concentration-dependent antioxidant capacity, with maximum efficacy at 30 μM (86.88 ± 2.318) which suggested that Vin had a potent antioxidant activity.

### Effects of Vinpocetine on Intracellular ROS levels in H_2_O_2_-treated and APAP-treated L02 cells

**Figure 2:**
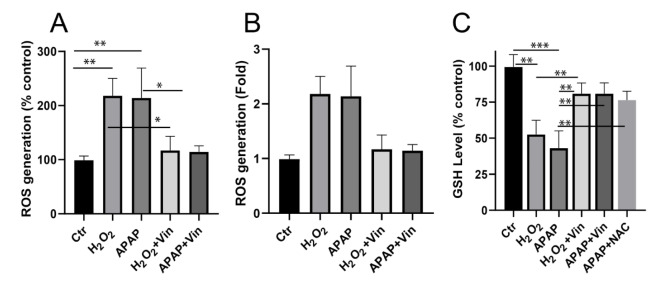
Effects of Vinpocetine on intracellular ROS contents and GSH level in H_2_O_2_-treated and APAP-treated L02 cells. A) Effects of Vinpocetine on intracellular ROS content. B) ROS generation fold increase. C) Effects of Vinpocetine on intracellular GSH content in H_2_O_2_-treated and APAP-treated L02 cells. Ctr; control, H_2_O_2_; 0.5 mM, APAP; paracetamol at 5 mM, Vin; Vinpocetine (20 µM). The data was shown as means ± SD, n=3. **P < 0.01, *P < 0.05, compared with indicated group

The intracellular ROS contents were depicted as a percentage of control and fold increase (Figure 2A-B(Fig. 2)). As shown in Figure 2A(Fig. 2), ROS contents in both H_2_O_2_-treated and APAP-treated L02 cells were increased to 218.0 ± 32.13 (2.18 fold) and 214 ± 55 (2.14 fold) compared to control (P < 0.01), respectively. Treatment with Vin (20 μM) resulted in ROS content of 117 ± 26 and 114 ± 11, respectively and compared with model group, P < 0.1.

### Effects of Vinpocetine on GSH level in H_2_O_2_-treated and APAP-treated L02 cells

The intracellular GSH contents (mg/g tissue) were calculated as a percentage of control (Figure 2C(Fig. 2)). As shown in Figure 2C(Fig. 2), GSH content in both H_2_O_2_-treated and APAP-treated L02 cells was decreased to 52 % ± 9 and 43 % ± 12, respectively, compared with control (P < 0.01). While treatment of cells with Vin restored the normal level of GSH content in both models, 80 % ± 7 and 81 % ± 6, respectively. NAC was used as positive control. Treatment of cells with NAC restored the level of GSH to 76 % ± 6, similar to Vin antioxidant activity.

### Cytoprotective activity of Vinpocetine against H_2_O_2_ and APAP-cytotoxicity

**Figure 3:**
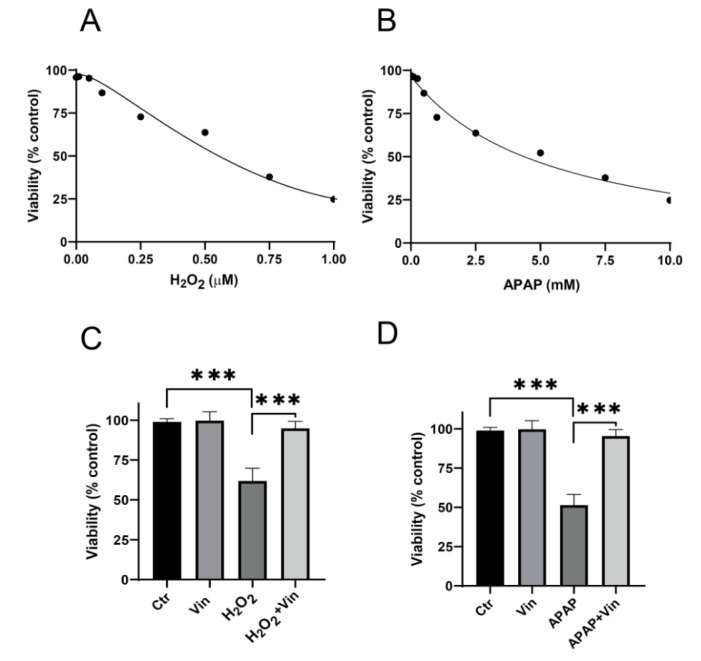
Cytoprotective activity of Vinpocetine against H_2_O_2_ and APAP-cytotoxicity. A) Cytotoxic effects of H_2_O_2_ on L02 cells, after 24 hrs treatment. B) Cytotoxic effects of APAP on L02 cells. Endpoint was 24 hrs. C) Cytoprotective activity of Vinpocetine against H_2_O_2_-induced cytotoxicity. D) Cytoprotective activity of Vinpocetine against APAP-induced cytotoxicity. Ctr; control, H_2_O_2_; 0.5 mM, APAP5; Paracetamol at 5 mM, Vin; Vinpocetine (20 µM). Data were presented as means ± SD, n=3. ***P < 0.001; indicated group

Effects of Vin on the viability of H_2_O_2_- treated, and APAP-treated L02 cells were also evaluated by MTS. The results are shown in Figure 3A and B(Fig. 3), where it reveals that H_2_O_2_ and APAP induced cytotoxicity in concentration dependent manner with IC_50 _equal 0.56 mM (H_2_O_2_) and 5.4 mM (APAP) (Figure 3A-B(Fig. 3)). Concurrent administration of Vin (20 μM) shows a significant increase in viability of both H_2_O_2_-treated (Figure 3C(Fig. 3)) and APAP-treated (Figure 3D(Fig. 3)) cells up to 94 %, 95 %, respectively.

### Effect of Vinpocetine on APAP-induced downregulation of NRf2-signaling pathway

**Figure 4:**
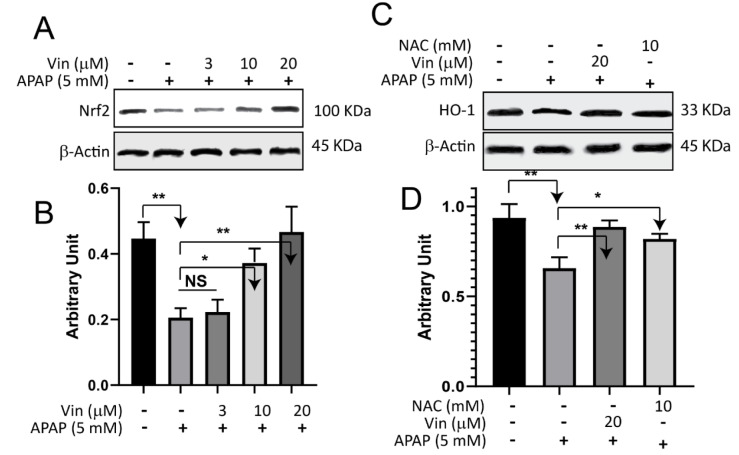
Effect of Vinpocetine on APAP-induced downregulation of NRf2-signaling pathway. A) L02 cells were treated with indicated compounds for 18 hrs, subsequently cell lysate subjected to immunoblotting technique and blotted against Nrf2 protein. B) Quantification of Western Blot band intensity of Nrf2 protein. C) L02 cells were treated with indicated compounds for 18 hrs, and then cells lysate subjected to Immunoblotting technique and blotted against HO-1 protein. D) Quantification of western blot band intensities of HO-1 protein

We utilized only APAP in this experiment as it is an approved drug in the clinic. As shown in Figure 4(Fig. 4), the expression of Nrf2 in the APAP-treated group dropped (0.2 ± 0.02 arbitrary unit) significantly (P < 0.05) when compared with control (0.45 ± 0.05 arbitrary unit). After being treated with escalated concentrations of Vin (3, 10, and 20 μM), the relative protein levels of Nrf2 were restored in a concentration-dependent manner (Figure 4A, B(Fig. 4)), to 0.22 ± 0.03, 0.38 ± 0.04 (P < 0.05), and 0.47 ± 0.07 (P < 0.01), arbitrary unit, respectively, compared with APAP-treated group. In a similar pattern, the relative protein level of HO-1 (Figure 4C(Fig. 4)) was significantly dropped (0.65 ± 0.06) in the APAP-treated group when compared with control (0.9 ± 0.07, P < 0.05). Cells, after being treated with Vin or NAC (as positive control) of 20 μM and 10 mM, respectively, the relative protein expression of HO-1 was upregulated to 0.89 ± 0.04 (P < 0.01) and 0.81 ± 0.02 (P < 0.05), respectively, compared with the APAP-treated group 0.65 ± 0.06, arbitrary unit.

### Molecular docking approach of Vinpocetine against Keap-1 protein

**Figure 5:**
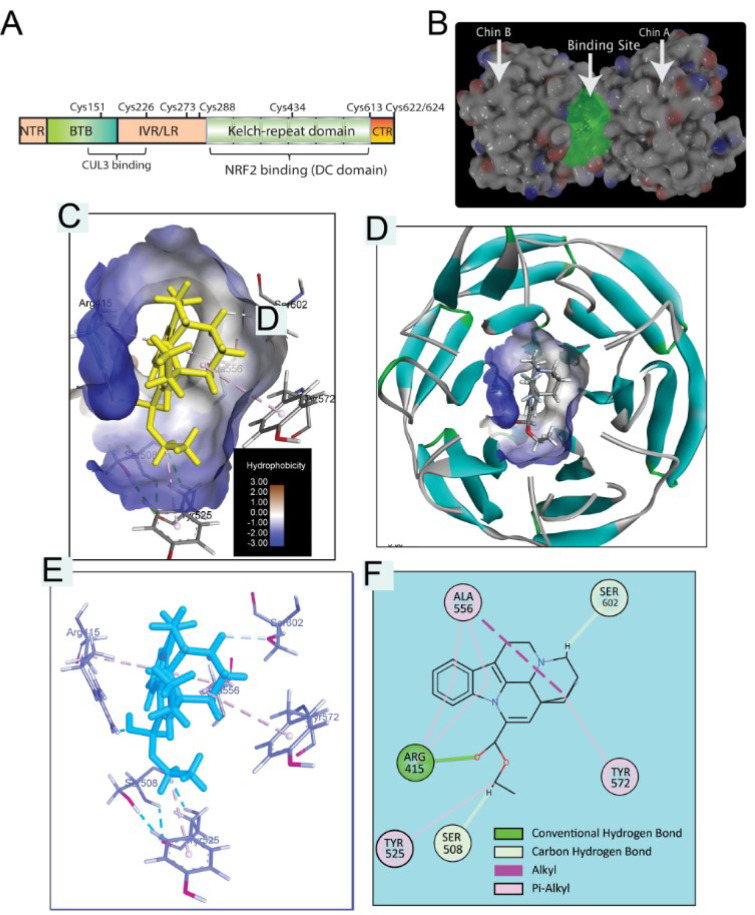
Molecular docking analysis of Vinpocetine-Keap1 (4L7B) interaction. A) Structural basis of Keap1 interactions with Nrf2. B) Crystal structure of Keap1 (4L7B) showing chain A, chain B and highlighted binding pocket. C) Vinpocetine in binding site of Keap1 (hydrophobicity protein surface). D) Vinpocetine and binding pocket inside Keap1 protein helix presentation (protein secondary structure). E) Vinpocetine Keap1 interaction in 3D presentation. F) Vinpocetine Keap1 interaction in 2D presentation.

To further elucidate the possible binding site between Vin and Keap1, docking simulation was carried out (Figure 5(Fig. 5)). As shown in Figure 5C-D(Fig. 5), Vin strongly binds into the binding pocket of chain B with binding energy -6.6 kcal/mol and was surrounded by amino acid residues, such as Y334, S363, R380, N414, R415, S508, S555, Y572, and S602, which is the binding domain as previously reported (Jnoff et al., 2014[8]). Moreover, the detail of binding mode of Vin to Keap1 is shown in Figure 5(Fig. 5) and summarized in Table 1(Tab. 1). Molecular interactions between Vin and Keap1 consisted of hydrogen bonds as alkyl and Pi-alkyl interactions (Figure 5E-F(Fig. 5) and Table 1(Tab. 1)). The hydrogen bonding interactions between Vin and ARG415, SER508 and SER605 are shown with the distant as 1.96, 3.1 and 2.44, 2.75 Å, respectively (Figure 5E-F(Fig. 5) and Table 1(Tab. 1)). As shown in Figure 5E-F(Fig. 5), Pi-alkyl interactions are established between Vin and ALA556, ALA556, ARG415, ARG415, TYR525, and TYR572 residues, in addition Alkyl interaction between Vin and ALA556.

## Discussion

Oxidative stress plays an essential role in the pathogenesis of various hepatic diseases, while the supplementation of compound with antioxidant activity might be a strategy to prevent or mitigate oxidative stress-related liver injuries (Costantini, 2019[5]; Li et al., 2015[11]). Vinpocetine (Vin) is a synthetic alkaloid. Vin has been used in the treatment of central nervous system disorders for decades. It has been established that Vin can mitigate and prevent many stress-related diseases (Zhang et al., 2018[20]; Zhang and Yang, 2014[19]; Patyar et al., 2011[14]; Medina, 2010[12]; Jeon et al., 2010[7]; Al-Kuraishy et al., 2020[2]).

In the present study, we demonstrated that Vin is non-toxic to L02 cells and has a potent antioxidant activity. Additionally, Vin suppressed H_2_O_2_- and APAP-induced ROS production, and elevated intracellular antioxidant GSH contents, leading to cytoprotective activities against H_2_O_2_- and APAP-induced hepatotoxicity in a normal human hepatic cell line, L02 cells.

Nrf2 is considered a “critical transcription factor” that orchestrates multiple downstream antioxidant enzymes such as HO-1, NQO-1 and GCLC (Glutamate-cysteine ligase catalytic subunit). These key players are involved in many cellular functions like drug biotransformation, and antioxidative buffering system. Upon nuclear translocation of Nrf2, it upregulates synthesis of GSH (non-enzymatic antioxidant protein), which play a critical role in elimination of intracellular ROS induced by varieties of drugs and chemical toxicants (Li et al., 2015[11]; Costantini, 2019[5]; Surh and Na, 2008[17]), such as APAP and H_2_O_2_. Our results demonstrated that Vin induced upregulation of Nrf2 expression in dose-dependent manner by immunoblot analyses. The functionality of Vin as transcription factor and its translocation into nucleus was confirmed by upregulation HO-1 protein expression (enzymatic down-stream-antioxidant). GSH, a non-enzymatic downstream-antioxidant of Nrf2, plays an essential role in protecting hepatocytes against oxidative injuries. Under cellular oxidative stress conditions, GSH scavenge toxic radicals, which is oxidized to GSSG under GSH-Px enzymes (Li et al., 2015[11]; Kachadourian et al., 2011[9]; Costantini, 2019[5]). The recovery of GSH level contributes to relieve and mitigate the pathological lesions, which attributed to oxidative stress injuries. Biochemical analysis showed that treatment of L02 cells with Vin elevated the GSH level, which were depleted by H_2_O_2_ and APAP. HO-1 is an inducible antioxidant enzyme, which is directly involved in the degradation of heme, as well as carbon monoxide and biliverdin.HO-1 is one of Nrf2 downstream-antioxidative enzyme that has implicated in the protection of cells from injury and cytotoxicity triggered by oxidative stress. Therefore, the chemicals with “HO-1 inducing activity” are considered to have a potential therapeutic cytoprotective activity. In this study, we demonstrated that treatment of hepatic cells with Vin prevented APAP-induced downregulation of HO-1. This study indicated that the protective effect of Vin against APAP-induced cytotoxicity was associated with Nrf2 activation and subsequently upregulation of its enzymatic and non-enzymatic antioxidant target genes, such as HO-1 and GSH respectively.

Keap1 acts as “substrate adaptor protein for the cullin-3 (Cul3)” that recruits Nrf2 to the “Cul3-Keap1-E3 ubiquitin ligase complex” and negatively regulating its activity, under normal conditions. Increasing evidence demonstrated that the interference of the interaction between Keap1 and Nrf2 is leading to the stabilization of Nrf2 by hampering ubiquitination process and proteasomal degradation. with enhancing nuclear translocation of Nrf2 (Unoki et al., 2020[18]; Silva-Islas and Maldonado, 2018[16]; Sharath Babu et al., 2017[15]; Costantini, 2019[5]; Bresciani et al., 2017[4]; Komatsu et al., 2010[10]). Thus, negatively targeting the Nrf2 binding site of Keap1 is considered as an attractive approach for the governing of Nrf2 activation and increasing cellular antioxidant capability. Our results showed that Vin could stabilize Nrf2 in L02 cells, as indicated by increasing the level of Nrf2 expression protein in Vin-treated group compared to APAP group. Molecular docking analysis indicated that upregulation of Nrf2 protein expression by Vin was attributed to the interference of protein-protein interaction between Nrf2 and Keap1. Moreover, short distance (1.96-2.75 Å), “shape-driven interactions” and hydrogen-binding interactions with vin, were playing essential roles in the disturbance of the binding capability between Keap1 and Nrf2 leading to the stabilization of Nrf2. Therefore, this docking approach suggested a reasonable-binding model of the Vin-Keap1, that may provide a profound description and rational understanding of the interactions between vin and Keap1 protein.

**Figure 6:**
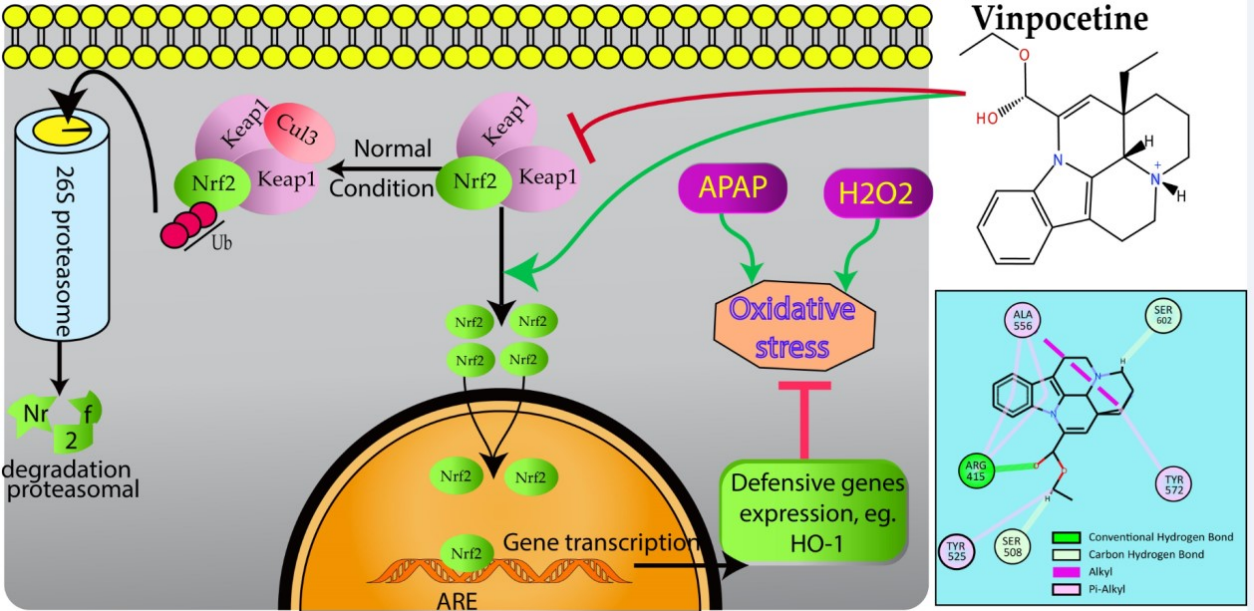
Schematic presentation of probable protective mechanism of Vinpocetine against oxidative stress injury in L02 cells. Vinpocetine directly disrupts Keap1 binding to Nrf2 to activate the Nrf2-ARE pathway and further attenuate H_2_O_2_ and APAP-induced oxidative stress damage

In conclusion, Vin effectively protects L02 cells against H_2_O_2_- and APAP-induced hepatotoxicity. This cytoprotective property of Vin is attributed to induction of Nrf2-mediated antioxidative response via the activation of HO-1 and restoration of normal GSH level, and disruption of protein-protein interactions between Keap1 and Nrf2 (Figure 6(Fig. 6)). Our findings suggest that Vin might be developed as Vin-based combination therapy against chemicals/drugs that induce oxidative stress-related liver damage, e.g. APAP (employing Vin as adjuvant therapy with the APAP drug regimen). 

## Conflict of interest

The authors have no conflicts of interest to declare.

## Figures and Tables

**Table 1:**
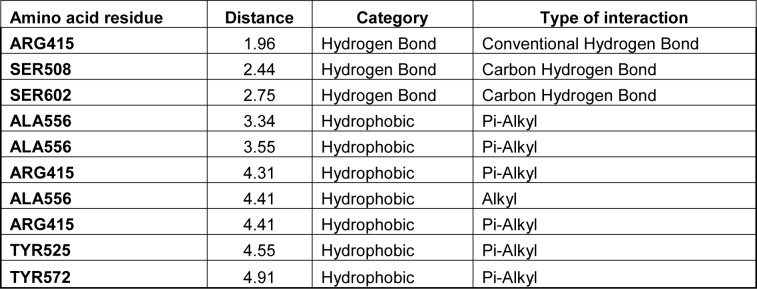
Molecular docking analysis of Vinpocetine-Keap1 interaction
